# Total neoadjuvant therapy based on short-course radiotherapy versus standard long-course chemoradiotherapy for locally advanced rectal cancer: a systematic review and meta-analysis of randomized controlled trials

**DOI:** 10.3389/fonc.2024.1515756

**Published:** 2024-12-24

**Authors:** Wenji Pu, Wenqi Chen, Haiman Jing, Jishi Li, Yong Jiang, Shasha Li, Weijie Wen, Zhiyuan Xu, Jing Jin

**Affiliations:** ^1^ Department of Clinical Oncology, The University of Hong Kong-Shenzhen Hospital, Shenzhen, China; ^2^ Medical Department of Shenzhen University, General Hospital of Shenzhen University, Academy of Clinical Medicine of Shenzhen University, Shenzhen, China; ^3^ National Cancer Center/National Clinical Research Center for Cancer/Cancer Hospital & Shenzhen Hospital, Chinese Academy of Medical Sciences and Peking Union Medical College, Shenzhen, China

**Keywords:** locally advanced rectal cancer, short-course radiotherapy, consolidation chemotherapy, total neoadjuvant therapy, neoadjuvant chemoradiotherapy, pathological complete response, overall survival, disease free survival

## Abstract

**Background:**

We conducted the meta-analysis to compare the therapeutic effects of total neoadjuvant therapy (TNT) based on short-course radiotherapy followed by consolidation chemotherapy (SCRT/CCT) and long-course chemoradiotherapy (LCCRT) for locally advanced rectal cancer (LARC) according to certain significant randomized controlled trials (RCTs).

**Methods:**

The researchers retrieved several databases, including PubMed, Embase, Web of Science, and the Cochrane Library, to collect all the relevant literature published since the establishment of the databases until July 30, 2024, and then screened to determine the qualified literature and extracted the relevant information. Finally, RevMan 5.4 software was utilized to conduct the meta-analysis for determining the 95% confidence interval (CI) and pooled risk ratio (RR). There were 9 study indicators, including the pathologic complete remission (pCR) rate, tumor downstaging rate, R0 resection rate, sphincter preservation rate, disease-free survival (DFS), overall survival (OS), acute ≥3 grade toxicity rate, surgery complication rate, and distant recurrence rate. When moderate, even severe, heterogeneity was found, a random-effect model was applied; otherwise, a fixed-effect model was used for the analysis.

**Results:**

A total of 6 eligible RCTs and 2259 participants were included in this meta-analysis. Compared with the standard LCCRT, TNT treatment on the basis of SCRT/CCT increased the pCR rate significantly [RR = 1.67, 95% CI (1.36, 2.04), P < 0.00001], especially in ≥ 4 cycles of the CCT arm [RR = 1.77, 95% CI: (1.41–2.23), p < 0.00001], and led to a similar tumor downstaging rate [RR = 0.99, 95% CI (0.85, 1.15), P = 0.92]. Moreover, survival outcomes, distant recurrence rate, and surgical indicators were comparable between the two groups.

**Conclusion:**

For LARC patients, the SCRT/CCT regimen not only has a higher pCR rate, equivalent OS, and comparable additional indicators versus standard LCCRT but also shortens the treatment time, costs less, and improves patients’ adherence to the innovative anti-tumor therapy; hence, with the concept of acute toxicity control, it could be further widely and safely utilized, especially in resource-limited settings.

**Systematic review registration:**

https://www.crd.york.ac.uk/prospero/, identifier CRD42024600180.

## 1 Introduction

In terms of incidence, colorectal cancer (CRC) is the third most common malignant tumor worldwide, with around 2.3 million cases. Its fatality rate has surpassed liver cancer to take the second position, skyrocketing to approximately 0.9 million deaths (1). As such, CRC is a serious threat to human health. About half of all CRC cases are of rectal cancer (RC), which is most common among individuals over 45 and has a far greater prevalence in men (2). In addition, China is predicted to have an enormous number of cases of CRC in 2022. According to the most recent data (3), China is estimated to have as many as 517,000 CRC cases (ranked second only to lung cancer) and 240,000 fatalities (ranked fourth), which is a concerning number.

About 60–70% of patients with RC develop locally advanced rectal cancer (LARC, which means TNM stage II-III RC), which is also staged as T3/T4 and/or including regional lymph node metastases (T3-4N0M0 or TanyN+M0); however, there is no universally accepted definition for it. For LARC, there currently exist two established preoperative neoadjuvant regimens: short-course radiotherapy (SCRT) with immediate surgery (widely applied in northern Europe) and conventional long-course chemoradiotherapy (LCCRT) based on fluorouracil with surgery 6–8 weeks later (accepted by the United States and southern Europe) (4). The efficacy and safety of the two strategies have already been demonstrated in prior studies (5–7), including decreasing tumor local recurrence and improving survival. However, in terms of pathologic complete remission (pCR) and potential micrometastasis (higher distant disease recurrence), SCRT appears to be less effective compared with LCCRT (8). Hence, SCRT is more suitable for low-to-moderate risk LARC (e.g., T3N0M0 and T1-2N1M0 without extramural vascular invasion, compromised mesorectal fascia, or positive circumferential resection margin) (2, 4). Moreover, the Stockholm III and Polish II trials (5, 9) have fortunately shown that advancing intensive systemic chemotherapy in the interim between SCRT and surgery has successfully increased pCR and tumor downstaging rates in comparison to LCCRT, which can also shorten the duration of treatment, reduce financial toxicity, and improve patients’ adherence to subsequent treatment.

This systematic review and meta-analysis aims to provide a deeper understanding of short-course radiotherapy followed by consolidation chemotherapy (SCRT/CCT) regimens by summarizing the available data about total neoadjuvant therapy (TNT) based on SCRT/CCT for LARC. It also investigates the efficacy of the included randomized controlled trials (RCTs) in terms of disease-free survival (DFS), overall survival (OS), pCR rate, surgical R0 resection rate, and other metrics.

## 2 Material and methods

### 2.1 Inclusion criteria

According to the Population, Intervention, Comparison, Outcomes, and Study (PICOS) principle, the inclusion criteria were designed as follows: (1) Participants: patients with LARC without distant metastases confirmed by pathological and radiological examination; (2) Interventions: the preoperative neoadjuvant treatment regimen was SCRT (25 Gy/5 fractions) followed by several sequential cycles of consolidation chemotherapy (SCRT/CCT); (3) Comparisons: the standard neoadjuvant LCCRT regimen was adopted; (4) Outcomes: the inclusion of the literature contains at least one of the following 9 study indexes: pCR rate, tumor downstaging rate, R0 resection rate, sphincter preservation rate, DFS, OS, acute ≥3 grade toxicity rate, surgery complication rate, and distant recurrence rate; (5) Study design: only based on prospective RCTs aiming at ensuring the quality of our research.

### 2.2 Exclusion criteria

We systematically excluded the following publications: (1) studies involving LARC patients who have developed tumors in other sites and distant metastatic tumors, combined with severe cardiopulmonary diseases or other severe underlying diseases; (2) only SCRT was administered without consolidation chemotherapy in the test arm; (3) non-prospective RCTs, such as observational and retrospective studies, single-arm clinical trails, and case reports; (4) animal and cellular experiments; (5) neoadjuvant therapies using immunologic and biologic agents; (6) unoriginal research, e.g., conference reports, systematic reviews, meta-analyses, study protocols, letters, expert opinions; (7) repeated publications from the same group at different assessment times; (8) studies that lacked a complete set of important indicators for extracting the required data.

### 2.3 Literature search

We comprehensively searched PubMed, Embase, Web of Science, and the Cochrane Library by using Medical Subject Heading (MeSH) terms of “rectal cancer,” “short-course radiotherapy,” “chemotherapy,” “long-course chemoradiotherapy,” and their individual corresponding free terms with combinations of Boolean operators (AND, OR, NOT). References from original research articles and literature systematic reviews were also checked to make sure no pertinent study was missed. The language restriction for the inclusion of literature was English. The last search was updated up to July 30, 2024. After screening titles and abstracts, literature selection was conducted by two independent reviewers (Wenji Pu and Wenqi Chen). Besides, we reviewed the reference lists of relevant literature to confirm all eligible studies were included in our systematic review and meta-analysis, which has been registered in PROSPERO (the registration ID CRD42024600180).

### 2.4 Assessing the risk of bias of included studies

We performed a comprehensive quality assessment of all included RCTs using the Cochrane Collaboration’s risk for bias assessment tool, which covers random allocation method, allocation concealment, blinding, data completeness, selective reporting, and other possible sources of bias (10). For each item, three different evaluation levels were given: low risk, unclear, and high risk. The quality of the data was assessed by means of the Review Manager 5.4 software to map the quality evaluation. Two investigators should conduct a literature search and screening according to the inclusion and exclusion criteria, and in case of disagreement, agreement should be reached through negotiation; if a dispute still exists after negotiation, a third reviewer should be referred to make the final decision.

### 2.5 Data extract

The following information was extracted from each selected publication, if available: first author, year of publication, baseline information of tumor patients, follow-up time, interventions and comparisons, pathological outcomes, tumor downstaging rate, survival and recurrence rates, acute toxicity, complications, and surgical procedures. In particular, pCR was defined as no residual tumor cells on pathological examination of resected tumor specimens after total mesorectal excision; R0 resection was defined as no tumor cells detected within the cut margins of the rectal specimen; and acute ≥3 grade toxicity rate was defined as serious adverse events occurring during preoperative neoadjuvant therapy or postoperative adjuvant chemotherapy.

### 2.6 Statistical analysis

All statistical analyses were performed using RevMan 5.4 software from the Cochrane Collaboration among the selected publications, if available. Heterogeneity of the literature data was assessed by I^2^ values and P values based on χ^2^ tests; if P ≥ 0.1 and I^2^ < 50%, it indicated that the heterogeneity was insignificant and a fixed-effect model was analyzed; otherwise, a random-effect model was employed. If necessary, potential sources of heterogeneity were explored by sensitivity analysis or subgroup analysis. Risk ratio (RR) was chosen as the effect value for statistical analysis for bivariate variables (the value greater than 1 means a higher rate of events in the former group), and P < 0.05 was considered a statistically significant difference. Finally, RevMan 5.4 software was utilized to calculate pooled RR and 95% confidence interval (CI).

## 3 Results

### 3.1 Study selection

**Figure 1:**
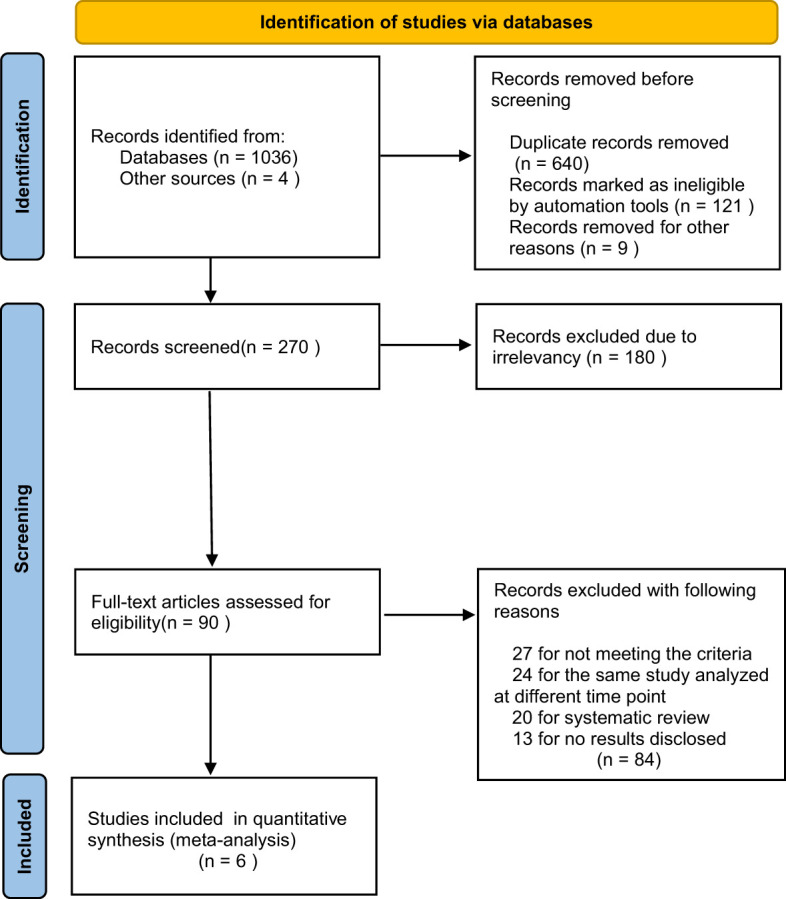
PRISMA 2020 flow diagram for systematic reviews which included searches of databases.

A total of 1040 relevant publications were searched, and 640 duplicates and 121 ineligible records were removed. After reviewing all the titles and abstracts, 180 of the publications were excluded due to irrelevancy. Then, 90 potentially eligible full-text articles were further assessed. We also excluded another 83 records, including 27 articles for not meeting the criteria, 24 articles for the same study analyzed at different time points, 20 articles for systematic review, and 12 articles for no results disclosed. Finally, we included 6 RCTs (9, 11–15) in our meta-analyses (the flow diagram is shown in **Figure 1**).

### 3.2 Characteristics of the included study

**Figure 2:**
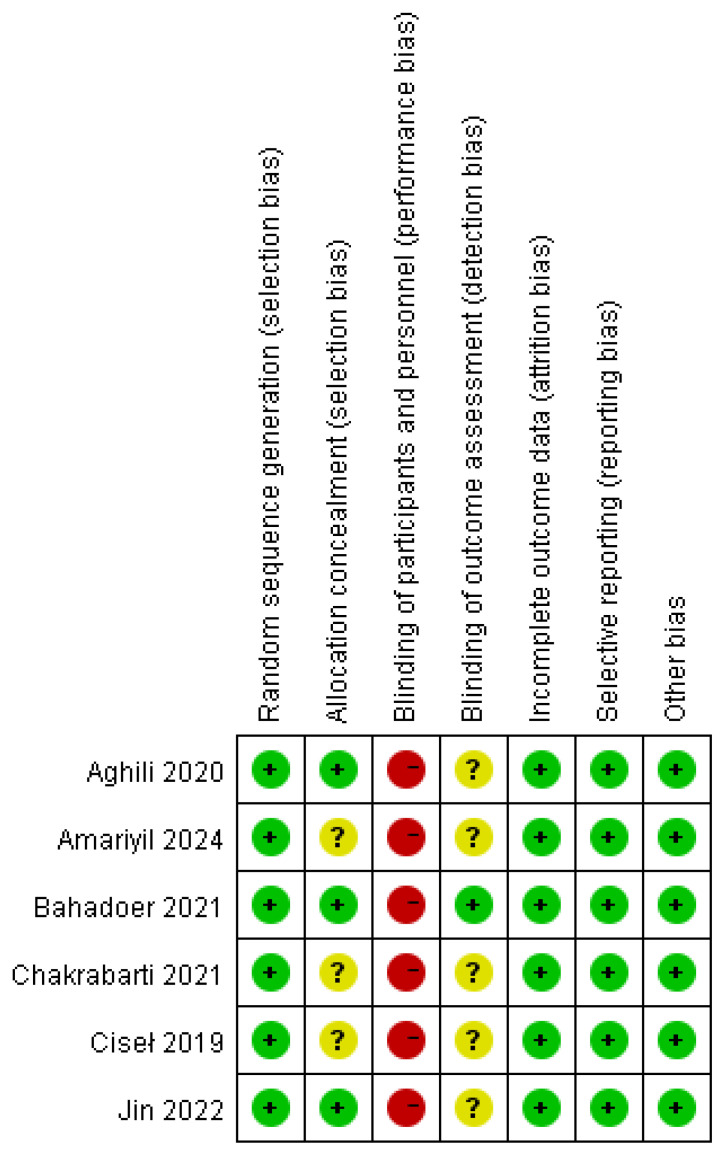
Risk of bias summary of the included RCTs.

**Figure 3:**
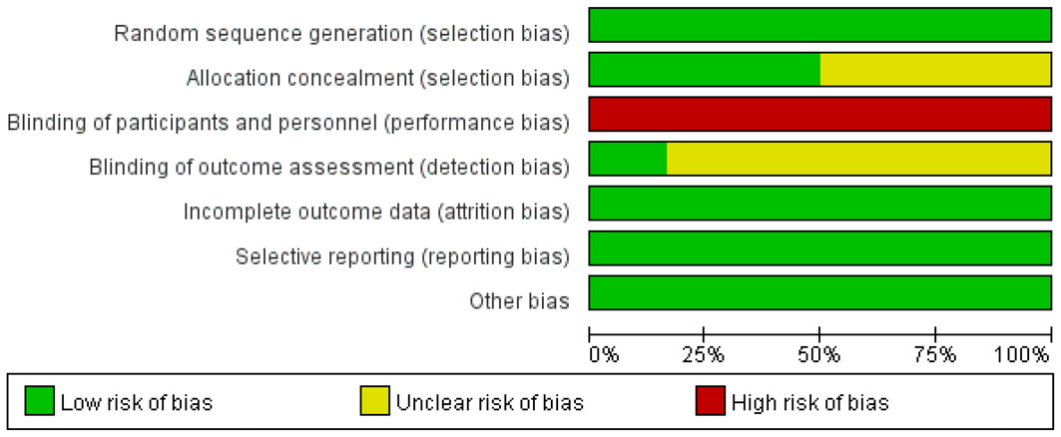
Risk of bias graph.

All of the involved LARC patients in the RCTs were informed of the treatment regimen at randomization. In addition, the risk of bias assessment and summary is illustrated in **Figures 2**, **3**. A total of 2259 LARC patients were assigned to the SCRT/CRT group (n = 1144) or LCCRT group (n = 1115). The tumor characteristics of studies are shown in **Table 1**, which demonstrate similarities between the two groups.

**Table 1:** Summary of the patients' characteristics of the included prospective studies.

References	Sample size	Year of enrollment	Staging	Interventions	Patient numbers (Man/woman%)	Average age (Years)	RT dose/fraction	CT regimen	Surgery interval (Weeks)	Follow-up time (Months)
Ciseł 2019 (9)	515	2008–2014	cT3-4M0	SCRT/CCT	261 (70%/30%)	60	25Gy/5F	FOLFOX × 3	12	84
				LCCRT	254 (66%/34%)	59	50.4Gy/28F	Infusion 5FU/LV + Oxaliplatin × 2	6	
Aghili 2020 (11)	60	2016–2020	II-III	SCRT/CCT	33 (55%/45%)	56	25Gy/5F	XELOX × 3-4	8	18
				LCCRT	27 (62%/38%)	53	50-50.4Gy/25-28F	XELODA, XELOX		
Bahadoer 2021 (12)	912	2011–2016	cT4a/b, cN2	SCRT/CCT	462 (65%/35%)	62	25Gy/5F	FOLFOX × 9 or CAPOX × 6	2-4	55
				LCCRT	450 (69%/31%)	62	50-50.4Gy/25-28F	XELODA	6-8	
Chakrabarti 2021 (13)	140	2017–2019	II-III	SCRT/CCT	69 (67%/33%)	42	25Gy/5F	CAPOX × 2	6-8	NR
				LCCRT	71 (66%/34%)	43	50-50.4Gy/25-28F	XELODA	8-12	
Jin 2022 (14)	599	2015–2018	II-III	SCRT/CCT	302 (72%/28%)	55	25Gy/5F	CAPOX × 4	6-8	35
				LCCRT	297 (70%/30%)	56	50Gy/25F	XELODA		
Amariyil 2024 (15)	33	2020–2021	cT3-4M0, or N+	SCRT/CCT	17 (47%/53%)	45	25Gy/5F	CAPOX × 3	8-10	NR
				LCCRT	16 (31%/69%)	46	45Gy/25F	XELODA		

NR, Not reported; RT, Radiotherapy; CT, Chemotherapy; SCRT/CCT, Short-course radiotherapy followed by consolidation chemotherapy; LCCRT, Long-course chemoradiotherapy.

### 3.3 Quality analysis

All of the included RCTs (9, 11–15) in our study reported the randomization process; nevertheless, LARC patients were informed regarding their treatment plan at allocation due to the difficulty in maintaining the radiotherapy regimen, a secret between the tumor patients and implementers. Nevertheless, the above restriction did not affect the outcome assessment.

### 3.4 Primary endpoint: pCR rate and tumor downstaging rate

**Figure 4:**
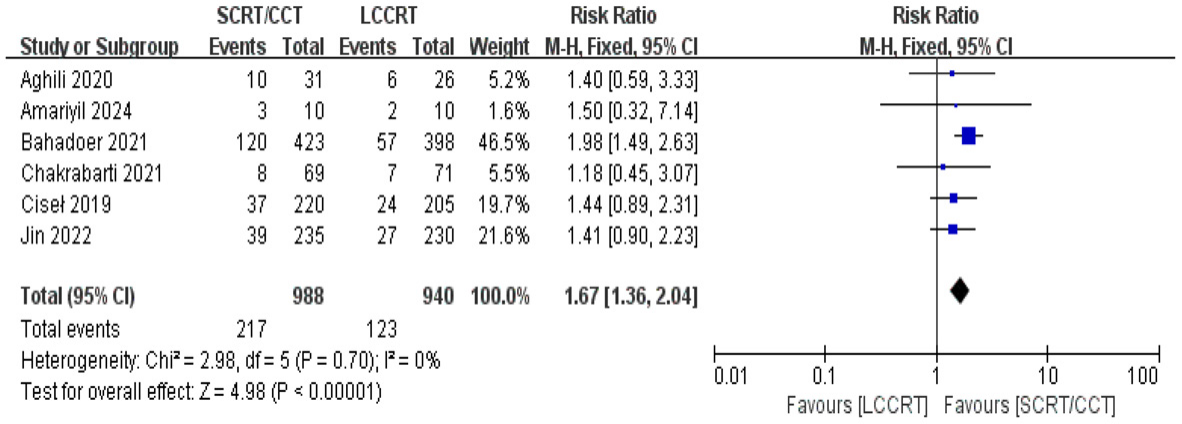
Forest plot for pCR rate.

**Figure 5:**
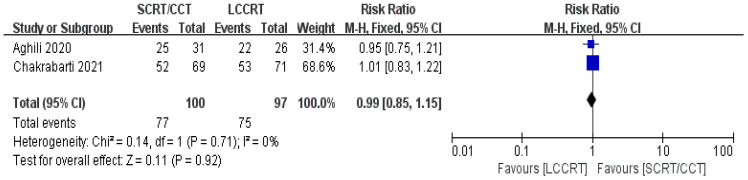
Forest plot for tumor downstaging rate.

The details of the study outcomes are summarized in **Table 2**. Pathological results commonly include the tumor downstaging rate, ypTNM stage, and pCR rate. The most widely used indicators in clinical trial design are pCR rate and tumor downstaging rate among the aforementioned metrics. All six trials (9, 11–15) were available for pCR comparison in our meta-analyses. As can be seen in **Figure 4**, there was a clear difference in the pCR rate between the SCRT/CCT group and the standard LCCRT group (RR = 1.67, 95% CI (1.36, 2.04), P<0.00001; I^2^ = 0%; a fixed-effect model was applied). Yet, there was no significant difference between the two arms with regard to the tumor downstaging rate, which was reported in two RCTs (11, 13) comprising 197 LARC patients [RR = 0.99, 95% CI (0.85, 1.15), P = 0.92; I^2^ = 0%; a fixed-effect model was used, presented in **Figure 5**].

**Table 2:** Summary of the treatment outcomes from the included prospective studies.

Study	Interventions	PCR rate	Tumor downstaging rate	R0 resection rate	Sphincter preservation rate	OS(events/total)	DFS (events/total)	Acute≥3 grade toxicity rate	Surgery complication rate	Distant recurrence rate
Ciseł 2019 (9)	SCRT/CCT	17% (37/220)	NR	NR	50% (110/220)	137/261	115/261	NR	16% (42/261)	35% (91/261)
	LCCRT	12% (24/205)	NR	NR	49% (100/205)	126/254	107/254	NR	15% (38/254)	33% (83/254)
Aghili 2020 (11)	SCRT/CCT	32% (10/31)	81% (25/31)	100% (31/31)	100% (31/31)	NR	NR	15% (5/33)	18% (6/33)	NR
	LCCRT	23% (6/26)	85% (22/26)	96% (25/26)	96% (25/26)	NR	NR	19% (5/27)	11% (3/27)	NR
Bahadoer 2021 (12)	SCRT/CCT	28% (120/423)	NR	90% (382/423)	64% (269/423)	382/462	332/462	48% (219/460)	NR	NR
	LCCRT	14% (57/398)	NR	90% (360/398)	54% (214/398)	369/450	298/450	25% (109/441)	NR	NR
Chakrabarti 2021 (13)	SCRT/CCT	12% (8/69)	75% (52/69)	87% (60/69)	32% (13/41)	NR	NR	NR	36% (25/69)	NR
	LCCRT	10% (7/71)	75% (53/71)	90% (64/71)	35% (17/48)	NR	NR	NR	30% (21/71)	NR
Jin 2022 (14)	SCRT/CCT	17% (39/235)	NR	92% (215/235)	53% (124/235)	258/298	192/298	27% (79/298)	14% (33/235)	22% (65/302)
	LCCRT	12% (27/230)	NR	88% (202/230)	56% (129/230)	220/293	183/293	13% (37/293)	16% (36/230)	23% (67/297)
Amariyil 2024 (15)	SCRT/CCT	30% (3/10)	NR	NR	58% (7/12)	NR	NR	25% (5/16)	17% (2/12)	NR
	LCCRT	20% (2/10)	NR	NR	60% (7/11)	NR	NR	7% (2/17)	0	NR

NR, Not reported; PCR, Pathologic complete remission; OS, Overall survival; DFS, Disease-free survival.

### 3.5 Survival indicators: overall survival and disease-free survival

**Figure 6:**
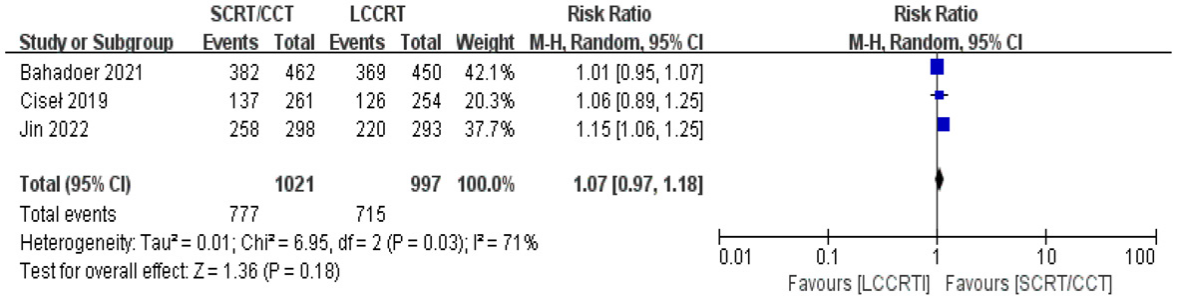
Forest plot for OS.

**Figure 7:**
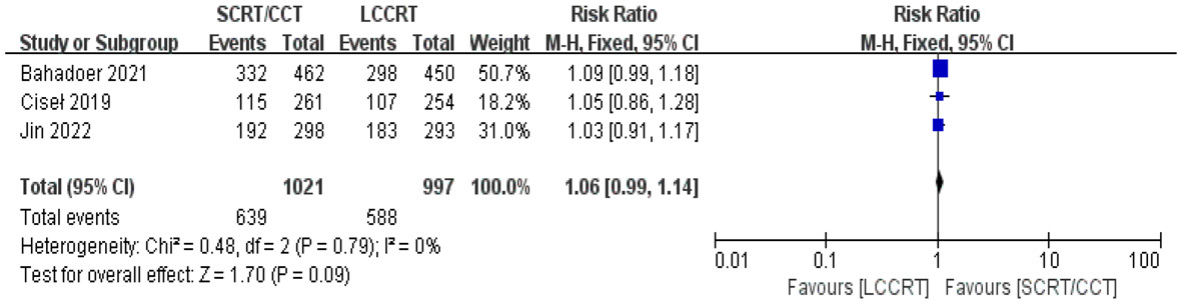
Forest plot for DFS.

In most cases, survival metrics have been regarded as an important reference to evaluate the effectiveness of clinical trials, such as OS and DFS. Survival data in our study, including OS and DFS, were analyzed when available at a fixed time point for assessment, with the median follow-up duration ranging from 6 to 84 months. Between the SCRT/CCT and LCCRT groups, there was no statistically significant difference in OS from the three available trials (9, 12, 14) [RR = 1.07, 95% CI (0.97, 1.18), P = 0.18; I^2^ = 71%; a random-effect model was employed to reduce heterogeneity error, shown in **Figure 6**]. With a total of 2018 patients, DFS was recorded in the three aforementioned investigations (9, 12, 14). However, there was no statistical difference between the two groups [RR = 1.06, 95% CI (0.99, 1.14), P = 0.09; I^2^ = 0%; a fixed-effect model was utilized, shown in **Figure 7**].

### 3.6 Surgical indicators: surgery R0 resection rate, sphincter preservation rate, and surgery complication rate

**Figure 8:**
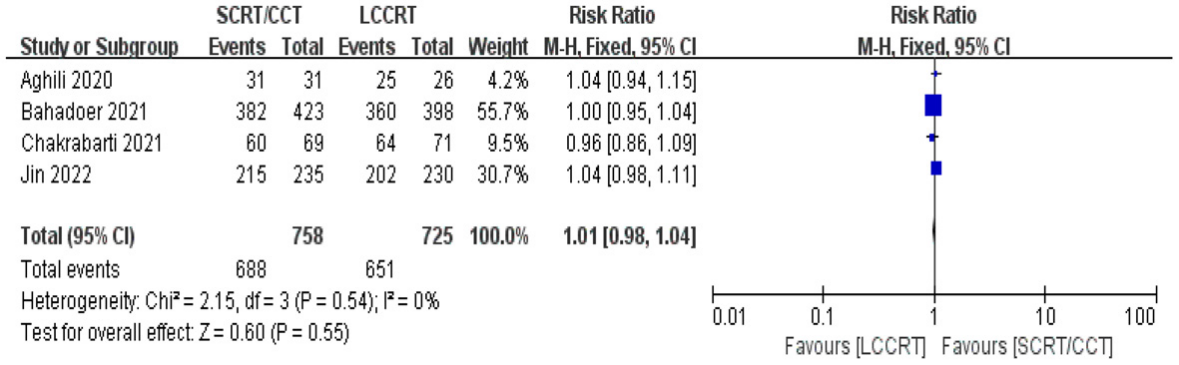
Forest plot for surgery R0 resection rate.

**Figure 9:**
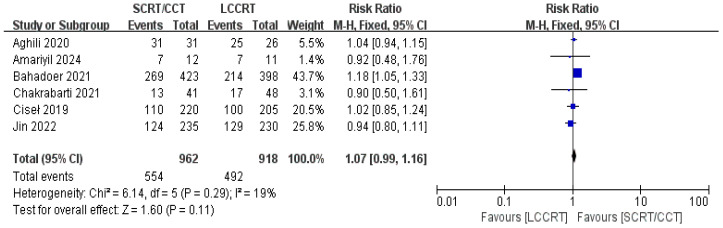
Forest plot for sphincter preservation rate.

**Figure 10:**
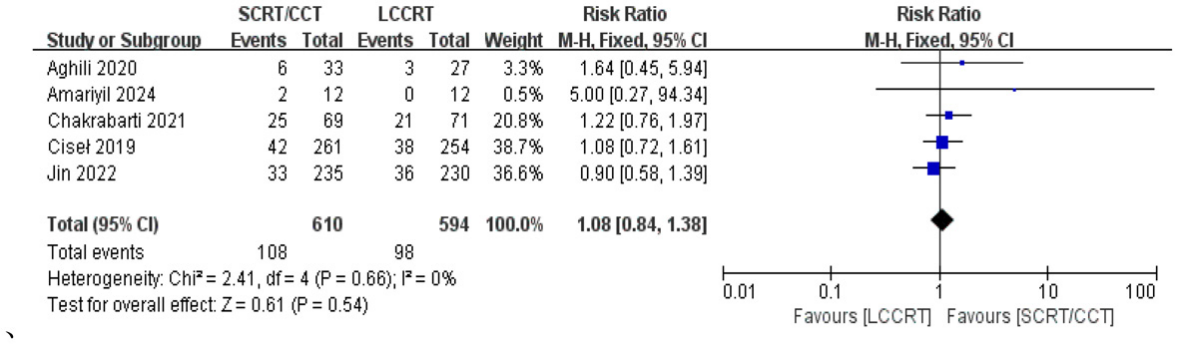
Forest plot for surgery complication rate.

4 RCTs (11–14) reported surgery R0 resection rates for assessment; there were similarities between the SCRT and LCCRT arms [RR = 1.01, 95% CI (0.98, 1.04), P = 0.55; I^2^ = 0%; a fixed-effect model was utilized, shown in **Figure 8**], which were close to borderline significance. In addition, regarding sphincter preservation rates, 6 trials (9, 11–15) enrolling 1880 patients offered data for assessment. SCRT/CCT had a similar sphincter preservation rate compared to LCCRT [RR: 1.07, 95% CI (0.99–1.16), P = 0.11; I^2^ = 19%; a fixed-effect model was exerted, shown in **Figure 9**]. Postoperative complications were defined as complications that occurred within 30 days after surgery resection. Moreover, there was no significant difference in the surgery complication rate between the two groups. As shown in **Figure 10**, the surgery complication rate in the standard LCCRT arm was comparable to the SCRT/CCT group [RR = 1.08, 95% CI (0.84, 1.38), P = 0.54; I^2^ = 0%; a fixed-effect model was applied].

### 3.7 Other indicators: acute ≥3 grade toxicity rate and distant recurrence rate

**Figure 11:**
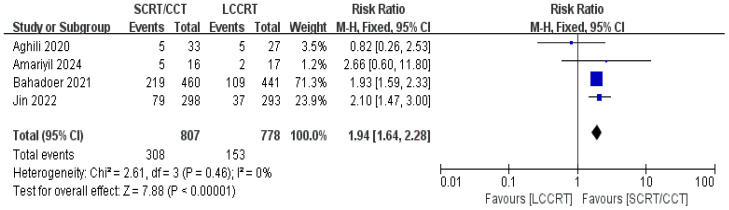
Forest plot for acute ≥3 grade toxicity rate.

**Figure 12:**
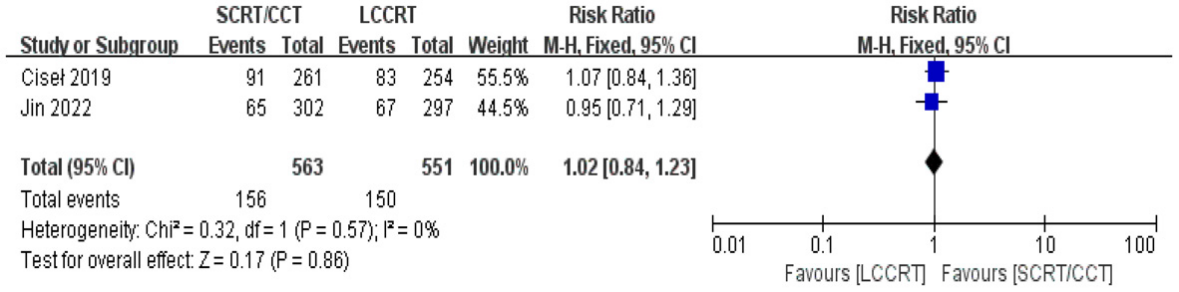
Forest plot for distant recurrence rate.

The sustainability of following treatment in tumor patients is reinforced when treatment-related toxicities, especially anticancer therapy toxicities, including acute ≥3 grade toxicity and late complications, are controlled below tolerable limits. Acute toxicity was mentioned in all of the included studies. According to the Common Terminology Criteria for Adverse Events (CTCAE), acute toxicity was classified. Based on the available data (11, 12, 14, 15), we only evaluated acute adverse events with a grade of ≥3. For instance, there was a significant difference in the acute ≥3 grade toxicity rate between the two groups. As seen in **Figure 11**, the toxicity rate was obviously higher in the SCRT/CCT group [RR = 1.94, 95% CI (1.64, 2.28), P<0.00001; I^2^ = 0%; a fixed-effect model was applied]. However, distant recurrence rates were reported in two RCTs (9, 14), which did not significantly differ between the SCRT/CCT and LCCRT cohorts [RR = 1.02, 95% CI (0.84, 1.23), P = 0.86; I^2^ = 0%; a fixed-effect model was exerted, shown in **Figure 12**].

### 3.8 Subgroup analysis according to < or ≥ 4 cycles of the consolidation chemotherapy

**Figure 13:**
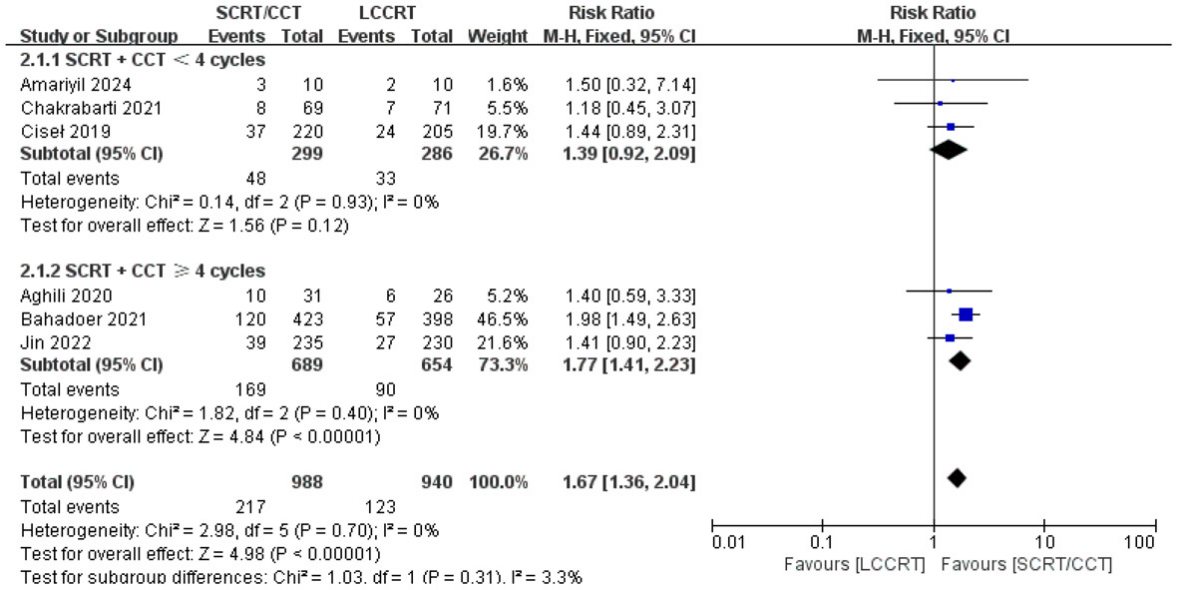
Forest plot for subgroup analysis of the pCR rate.

**Figure 14:**
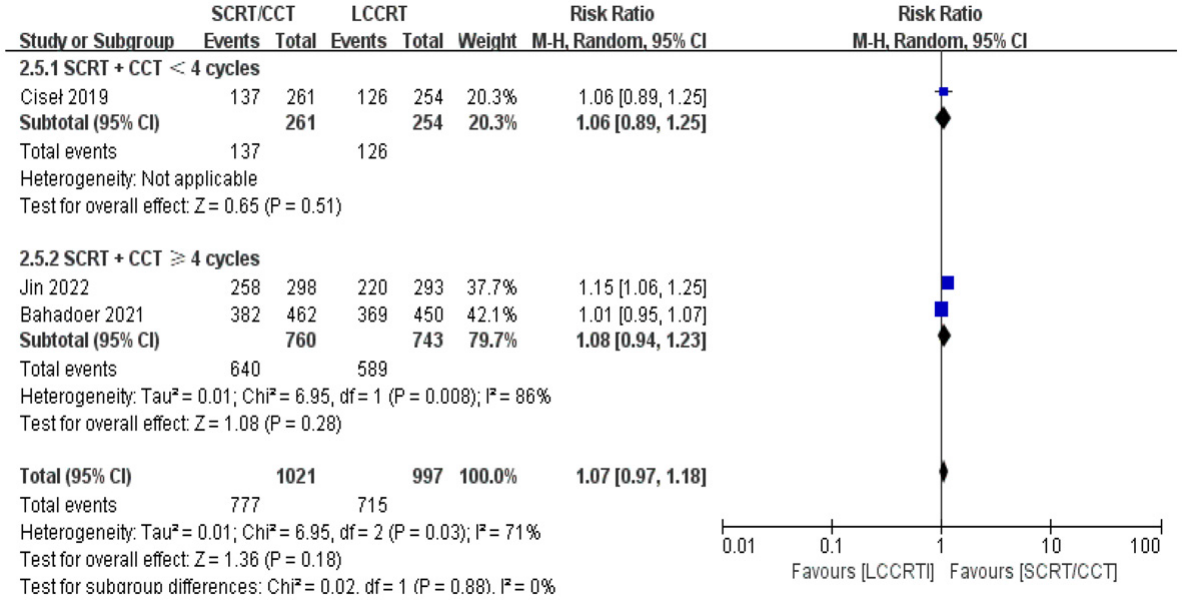
Forest plot for subgroup analysis of the OS.

**Figure 15:**
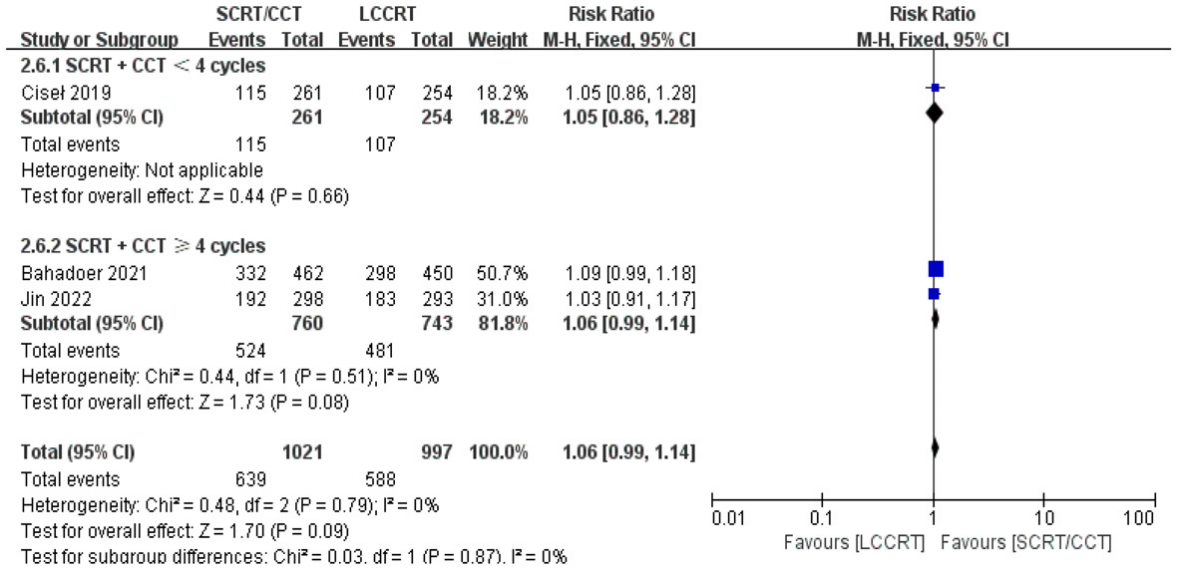
Forest plot for subgroup analysis of the DFS.

We investigated further with subgroup analysis (9, 11–15). With SCRT followed by at least 4 cycles of the CCT arm, the subgroup analysis revealed a significantly higher pCR rate than the LCCRT arm [RR = 1.77, 95% CI: (1.41–2.23), P < 0.00001]. On the other hand, given that the CCT was given in less than 4 cycles, there was no difference in pCR status between the two arms [RR = 1.39, 95% CI: (0.92–2.09), P = 0.12; I^2^ = 0%; a fixed-effect model was applied]. However, with respect to the CCT cycles on OS and DFS events, the CCT cycles ≥ 4 or < 4 subgroups both proved no superiority in the SCRT/CCT arm (**Figures 13**–**15** illustrate the above-mentioned results).

## 4 Discussion

Due to consolidation chemotherapy and prolonged surgery, the SCRT/CCT arm showed non-inferiority in tumor-downstaging status and had an enhanced pCR rate, according to our meta-analysis. Consequently, a trend of a lower distant recurrence rate was also noted. This is because SCRT may be more conducive to activating the systemic immune system and promoting local T-cell activation to maximize anti-tumor efficacy. However, these advantages did not translate into benefits for survival. The two arms performed comparably in terms of survival outcomes (OS and DFS events) but also surgical indicators (such as surgery R0 resection rate, sphincter preservation rate, and surgery complication rate). Oncologists should be made aware of the fact that the SCRT/CCT group experienced an increase in acute grade 3–4 adverse events, despite the fact that the pCR rate was increasing in that group.

Prior research (16, 17) has demonstrated improved OS and DFS in patients who achieve pCR following neoadjuvant therapy for LARC. PCR has been considered to be a highly valuable signal for evaluating the effectiveness of short-term treatment and predicting long-term prognosis, even though it cannot entirely replace OS or DFS. The results of this meta-analysis showed that the pCR rate in the experimental group was significantly higher than that in the control group [RR = 1.67, 95% CI (1.36, 2.04), P<0.001]. The administration of systemic chemotherapy in the experimental group and the extension of the time between chemoradiotherapy and surgery may be the reasons for the rise in the pCR rate. In conventional neoadjuvant therapy, the disadvantage of applying SCRT combined with immediate surgery for LARC patients is that the interval is too short and the possibility of clinical tumor complete remission is lost in most instances. The results of the Stockholm III trial (5) showed that if surgery was delayed for 4–8 weeks after SCRT, the pCR rate increased significantly to 11.8%, while the pCR rate in the group with immediate surgery after SCRT was only 2.1%. According to previous studies (18–20), the pCR rate after delayed surgery with SCRT, although increased, was not higher than that with LCCRT; therefore, simply extending the interval between SCRT and surgery is not an optimal substitute for conventional LCCRT for the treatment of LARC.

A prospective phase II trial conducted by Garcia-Aguilar et al. (21) compared the effect of SCRT followed by an additional 6 cycles of mFOLFOX6 with surgery in the treatment of LARC on tumor regression. The tumor regression rate improved significantly with increasing cycles of chemotherapy, with a 25% pCR rate in patients who were added 2 cycles of mFOLFOX6 and a 38% pCR rate in patients who were added 6 cycles of mFOLFOX6, which suggested that tumors may shrink if systemic chemotherapy is added to the interval between SCRT and surgery. Hence, more and more RCTs are looking at the feasibility and safety of consolidation or induction chemotherapy. This meta-analysis's improved pCR rate indicated that SCRT plus consolidation chemotherapy (≥ 4 cycles) could be a feasible treatment option for LARC with organ retention needs as well. This study also fully confirmed the hypothesis that the SCRT/CCT regimen would provide similar long-term survival outcomes compared to the LCCRT regimen. Moreover, the results of our meta-analysis indicated that the experimental group’s sphincter preservation rate was comparable to the control group’s, which suggests that more LARC patients may be able to achieve organ preservation, minimize the cost of treatment, and improve their quality of life by choosing the SCRT/CCT scheme. Most importantly, some patients who have experienced clinical complete remission as a result of TNT based on SCRT/CCT may be able to avoid surgery in favor of the “watch-and-wait” strategy (22, 23).

We can’t be entirely positive. Acute grade 3 or higher adverse events were more common in the test group, according to the meta-analysis’s findings, and the difference was statistically significant [RR = 1.94, 95% CI (1.64, 2.28), P<0.001]. The enhanced acute side effects of preoperative systemic chemotherapy could be the cause of this outcome. Similarly, preoperative mFOLFOX-6 consolidation chemotherapy was reported in some studies to improve tumor remission rates and DFS; however, there was a corresponding rise in the incidence of acute toxicity (21, 24). According to the Thavaneswaran et al. meta-analysis study (25), acute grade 3 or higher adverse events were more frequent in the SCRT/CCT group, which is consistent with the results of our study. This meta-analysis showed a higher incidence of acute grade 3-4 adverse events in the SCRT/CCT group, while the overall surgical complications, such as the common anastomotic fistula and incisional infections, were not significantly different between the two groups.

There was no statistically significant difference in the distant metastasis rate between the trial and control groups. We found that distant metastatic occurrences were managed in the SCRT/CCT arm, which is in accordance with the results of the most recent RAPIDO study update (26). To investigate the efficacy of the TNT strategy and to more precisely interpret existing data, longer follow-up and homogeneous clinical trials may be required. Similar to the results of some large clinical trials, including the Polish II trial (9) in Poland, the RAPIDO trial (12) in the Netherlands, and the STELLAR trial (14) in China, our meta-analysis’s outcomes also showed no statistically significant difference in OS and DFS between the two groups, but the statistics undoubtedly indicated a trend toward better OS and DFS in the experimental group. The findings should be interpreted cautiously, nevertheless, as there was some moderate heterogeneity in the OS investigation. The larger clinical trials are required to support more trustworthy conclusions about OS and DFS benefits.

The results of this meta-analysis revealed no discernible difference between the two groups in terms of the R0 resection rate and tumor downstaging rate. According to Park et al. (27), peripheral margin-negative patients had a better 5-year DFS among those who did undergo preoperative chemoradiotherapy (CRT) (88.9% vs. 55.5%), the same as not receiving preoperative CRT (82.8% vs. 54.7%). Positive postoperative pathologic margins also tended to indicate a poor prognosis. In the same way, a study found that individuals with postoperative pathologic stage ypT0-2 were more likely than those with ypT3-4 to benefit from postoperative adjuvant chemotherapy (28). Although the results of this meta-analysis showed no statistically significant difference, the trial arm showed a slight trend toward tumor-downstaging benefits [RR = 0.99, 95% CI (0.85, 1.15), P = 0.92]. Furthermore, we discovered that treatment compliance was higher among patients in the SCRT group. This might be because SCRT, which is divided into 5 fractions, requires a shorter treatment time than LCCRT. In addition to being more affordable and convenient, SCRT also minimizes treatment-related economic toxicity and shortens the number of days that LARC patients must stay in the hospital, particularly in China, where radiotherapy resources are relatively limited.

Unfortunately, since the above RCTs did not include local recurrence rate thoroughly, it was not examined in our meta-study. Indeed, given the low locoregional recurrence rate reported by the LARCT-US study (29) and the combined results of the Polish and STELLAR trials (9, 14), SCRT/CRT should be regarded as unquestionably effective, even though the increased locoregional recurrence rate following longer follow-up in the RAPIDO trial is still unclear. In my opinion, there were a number of factors that were worth taking into account. First of all, by employing the 5 × 5 Gy SCRT [a biological dose equivalent to 2 Gy per fraction from 31 Gy (for α/β = 10) to 36 Gy (for α/β = 5), which is significantly lower than the LCCRT arm] along with consolidation chemo, the experimental group postponed surgery for 40 weeks, and tumor fibrosis ultimately made surgery more difficult and then increased LRR. Secondly, the RAPIDO trial did not conduct a centralized evaluation of radiation plans, which may lead to inadequate dose coverage of the planned target volume and intended recurrences. At last, the RAPIDO trial failed to reach its main goal because a significant percentage of LARC in the experimental arm had high-risk features, which raised the LRR and may have also been caused by inappropriate SCRT.

Therefore, treatment for LARC cannot be generalized; we need to incorporate high-risk factors such as the presence of T4 disease, threatened mesorectal fascia, extramural vascular invasion, lateral or extramesorectal lymph node metastasis, N2, less than 5 cm from the anal verge, and circumferential resection margin positivity, which are all important stratification factors for treatment selections and inform the design of subsequent clinical studies. Likewise, genetic mutations and dMMR status may affect treatment efficacy. We also agree that for LARC patients with high-risk features for local recurrence rate, LCCRT may be preferred due to the poor bioequivalent dose of SCRT, given the results of the RAPIDO trial (30–33). Therefore, in future studies, we need to screen out these high-risk populations suitable for the LCCRT regimen. However, at present, due to the lack of high-level evidence, our meta-study provides important reference options for LARC, especially in healthcare resource-constrained regions.

Several limitations of the present meta-analysis must be taken into consideration. Firstly, only 6 RCTs were included, which means that the number of tumor patients involved was relatively small; secondly, surgery for LARC may ultimately present uneven treatment results because the tumor characteristics were not exactly the same and high spatiotemporal heterogeneity in each study remained; thirdly, surgical ability and proficiency from global healthcare organizations may lead to distinct outcomes; fourth, the completion of adjuvant chemotherapy, the cycles and regimens of consolidation chemotherapy, and the surgical intervals all varied, even in RCTs, which may be the cause of the outcome errors. Eventually, we anticipate that further large-sample and multicenter trials, as well as longer follow-up data in the aforesaid RCTs, will be available in the future to address the aforementioned issues and offer more convincing proof supporting the SCRT/CCT therapy option for LARC patients.

Last but not least, it’s critical to specify at the beginning of treatment whether the sufferer’s objective is pursuing organ preservation by a “watch-and-wait strategy” for the reason that there has now existed more data supporting LCCRT for LARC patients being evaluated for non-surgical management (34). In this regard, the University of Stanford also provides important therapeutic references regarding balancing risk factors for local versus distant recurrence (35). Besides, the ongoing ACO/ARO/AIO-18 trial, which randomly assigns patients to TNT regimens of SCRT versus LCCRT followed by chemotherapy, has been anticipated to provide much-needed clarification on this choice.

## 5 Conclusions

Although there may be a larger chance of acute grade 3-4 adverse events, the SCRT/CCT scheme offers patients with LARC a higher pCR rate and an equivalent survival prognosis when compared to LCCRT (34–36). Thus, as a neoadjuvant alternative therapy option for LARC, our RCTs-based meta-analysis study shows that SCRT/CCT is safe and effective, which not only decreases treatment time costs but also increases patients’ compliance to subsequent treatment and facilitates organ retention. The SCRT/CCT regimen deserves to be further utilized, especially in China and economically deprived areas.

## Data Availability

The original contributions presented in the study are included in the article/supplementary material. Further inquiries can be directed to the corresponding authors.
