# Corrigendum: Total neoadjuvant therapy based on short-course radiotherapy versus standard long-course chemoradiotherapy for locally advanced rectal cancer: a systematic review and meta-analysis of randomized controlled trials

**DOI:** 10.3389/fonc.2024.1552188

**Published:** 2025-01-14

**Authors:** Wenji Pu, Wenqi Chen, Haiman Jing, Jishi Li, Yong Jiang, Shasha Li, Weijie Wen, Zhiyuan Xu, Jing Jin

**Affiliations:** ^1^ Department of Clinical Oncology, The University of Hong Kong-Shenzhen Hospital, Shenzhen, China; ^2^ Medical Department of Shenzhen University, General Hospital of Shenzhen University, Academy of Clinical Medicine of Shenzhen University, Shenzhen, China; ^3^ National Cancer Center/National Clinical Research Center for Cancer/Cancer Hospital & Shenzhen Hospital, Chinese Academy of Medical Sciences and Peking Union Medical College, Shenzhen, China

**Keywords:** locally advanced rectal cancer, short-course radiotherapy, consolidation chemotherapy, total neoadjuvant therapy, neoadjuvant chemoradiotherapy, pathological complete response, overall survival, disease free survival

In the published article, there was an error in the affiliation of Jing Jin. Instead of “Department of Radiotherapy, National Cancer Center/National Cancer Clinical Medical Research Center/Shenzhen Hospital, Cancer Hospital of Peking Union Medical College, Chinese Academy of Medical Sciences, Shenzhen, China”, it should be “National Cancer Center/National Clinical Research Center for Cancer/Cancer Hospital & Shenzhen Hospital, Chinese Academy of Medical Sciences and Peking Union Medical College, Shenzhen, China”.

In the published article, there was an error in the **Funding** statement. “The author(s) declare financial support was received for the research, authorship, and/or publication of this article. This research was supported by grants from Sanming Project of Medicine in Shenzhen (SZSM202211017) and Shenzhen Key Medical Discipline Construction Fund(SZXK014).” The correct **Funding** statement appears below.

“The author(s) declare financial support was received for the research, authorship, and/or publication of this article. This study was supported by grants from Shenzhen Medical Research Fund (SMRF, C2301001), Shenzhen High-level Hospital Construction Fund, Sanming Project of Medicine in Shenzhen (SZSM202211030), Shenzhen Key Medical Discipline Construction Fund (SZXK013) and Shenzhen Clinical Research Center for Cancer (No.[2021]287).”

In the published article, there was an error. In the *Literature search* part, the personal pronoun was misused.

A correction has been made to *Literature search* part. This sentence previously stated:

“We comprehensively searched PubMed, Embase, Web of Science, and the Cochrane Library by using Medical Subject Heading (MeSH) terms of “rectal cancer,” “short-course radiotherapy,” “chemotherapy,” “long-course chemoradiotherapy,” and its individual corresponding free terms with combinations of Boolean operators (AND, OR, NOT).”

The corrected sentence appears below:

“We comprehensively searched PubMed, Embase, Web of Science, and the Cochrane Library by using Medical Subject Heading (MeSH) terms of “rectal cancer,” “short-course radiotherapy,” “chemotherapy,” “long-course chemoradiotherapy,” and their individual corresponding free terms with combinations of Boolean operators (AND, OR, NOT).”

The authors apologize for this error and state that this does not change the scientific conclusions of the article in any way. The original article has been updated.

